# Regulation of transcription elongation in response to osmostress

**DOI:** 10.1371/journal.pgen.1007090

**Published:** 2017-11-20

**Authors:** Andrea Silva, Santiago Cavero, Victoria Begley, Carme Solé, René Böttcher, Sebastián Chávez, Francesc Posas, Eulàlia de Nadal

**Affiliations:** 1 Cell Signaling Research Group, Departament de Ciències Experimentals i de la Salut, Universitat Pompeu Fabra (UPF), E-08003 Barcelona, Spain; 2 Instituto de Biomedicina de Sevilla (IBiS), Hospital Virgen del Rocío-CSIC-Universidad de Sevilla, and Departamento de Genética, Universidad de Sevilla, Sevilla, Spain; Harvard Medical School, UNITED STATES

## Abstract

Cells trigger massive changes in gene expression upon environmental fluctuations. The Hog1 stress-activated protein kinase (SAPK) is an important regulator of the transcriptional activation program that maximizes cell fitness when yeast cells are exposed to osmostress. Besides being associated with transcription factors bound at target promoters to stimulate transcriptional initiation, activated Hog1 behaves as a transcriptional elongation factor that is selective for stress-responsive genes. Here, we provide insights into how this signaling kinase functions in transcription elongation. Hog1 phosphorylates the Spt4 elongation factor at Thr42 and Ser43 and such phosphorylations are essential for the overall transcriptional response upon osmostress. The phosphorylation of Spt4 by Hog1 regulates RNA polymerase II processivity at stress-responsive genes, which is critical for cell survival under high osmostress conditions. Thus, the direct regulation of Spt4 upon environmental insults serves to stimulate RNA Pol II elongation efficiency.

## Author summary

Cells coordinate various intracellular activities in response to sudden changes in the environment to maximize survival and cell fitness. Clear examples of this are the regulation of gene expression and cell cycle progression. Stress-Activated Protein Kinase (SAPK) signaling pathway acts as the central core for these responses. Major changes in gene expression are associated with cell exposure to environmental changes and several aspects of mRNA biogenesis appear to be targeted by SAPKs. Here, we describe a new cellular mechanism to tightly regulate the expression of the stress-dependent genes. Our data demonstrate that the SAPK signaling pathway impacts on core elements of the cellular machinery and directly targets elongating RNA Pol II by phosphorylating the Spt4 elongation factor. This novel finding changes the framework for the understanding of how cells modulate the transcriptional elongation process to increase gene expression efficiency and maximize cell fitness in response to external cues.

## Introduction

Cells sense and robustly respond to environmental fluctuations to maximize cell fitness. An increase in extracellular osmolarity provokes immediate cellular responses that are crucial for cell survival. These responses are mainly controlled by the High Osmolarity Glycerol (HOG) pathway [1,2] in which the p38-related Hog1 Stress-Activated Protein Kinase (SAPK) modulates almost any aspect of the cell physiology essential for cell survival under stress.

In response to osmostress, there is a major change in the transcriptional pattern of the cell that is crucial for long term adaptation [3,4]. The coordinated expression of such stress-responsive gene reprogramming is accomplished by the regulation of several steps in mRNA biogenesis and mRNA fate through the Hog1 SAPK (reviewed in [5,6]). This signaling kinase associates with chromatin [7,8] to specifically target RNA Pol II to stress-responsive genes in order to bypass the general down-regulation of gene expression that occurs during stress conditions [4,9]. Association of Hog1 with stress-responsive genes is strongly correlated with chromatin remodeling and increased gene expression [4,10–13]. The mechanisms by which Hog1 regulates initiation of transcription are well-described and involve direct phosphorylation of specific transcription factors [14,15] as well as recruitment of coactivators to osmo-responsive promoters [8,16,17] and chromatin modifying activities [12,18–20]. Hog1 also associates with the coding regions of osmo-dependent genes, where it acts as a transcription elongation factor specific for stress-responsive genes [21]. However, how this SAPK regulates elongation of stress-dependent genes still remains to be elucidated.

Transcription elongation by RNA Pol II is a dynamic and highly regulated step in the gene expression cycle, where elongation accessory and/or chromatin remodeling factors play an essential role (reviewed in [22,23]). The Spt5/Spt4 complex is a universally conserved RNA Pol II-associated factor with a pervasive role in transcription elongation ([24,25] and reviewed in [26]). Whereas Spt4 is a small zinc finger protein, Spt5 is a large multi-domain protein consisting of an N-terminal acidic domain, a NusG N-terminal (NGN) domain, multiple Kyprides-Ouzounis-Woese (KOW) domains and a set of short repeats at its C-terminus (CTR). Crystallographic studies have shown that Spt4 interacts intimately with the NGN domain of Spt5 in formation of the Spt4-Spt5 heterodimeric complex that serves to stimulate polymerase processivity [27–31]. Apparently, Spt4 might stabilize the elongating RNA Pol II, thereby allowing polymerase molecules to travel long distances through a gene without dissociating from the template [32]. Spt4 is also required for the transcription of GC-rich DNA sequences [33] and it has been shown to help RNA Pol II to traverse lengthy gene regions that encode polyglutamine repeats [34].

Here, we show that in response to stress the Hog1 SAPK phosphorylates Spt4 to regulate the transcriptional response. The targeting of Spt4 by Hog1 enhances RNA pol II processitivity at stress-responsive genes. Remarkably, the non-phosphorylatable mutant of Spt4 renders cells partially osmo-sensitive. Therefore, our results demonstrate that the HOG signaling pathway directly targets elongating RNA Pol II to modulate polymerase processivity in response to environmental insults.

## Results

### The Hog1 SAPK directly phosphorylates Spt4

**Fig 1:**
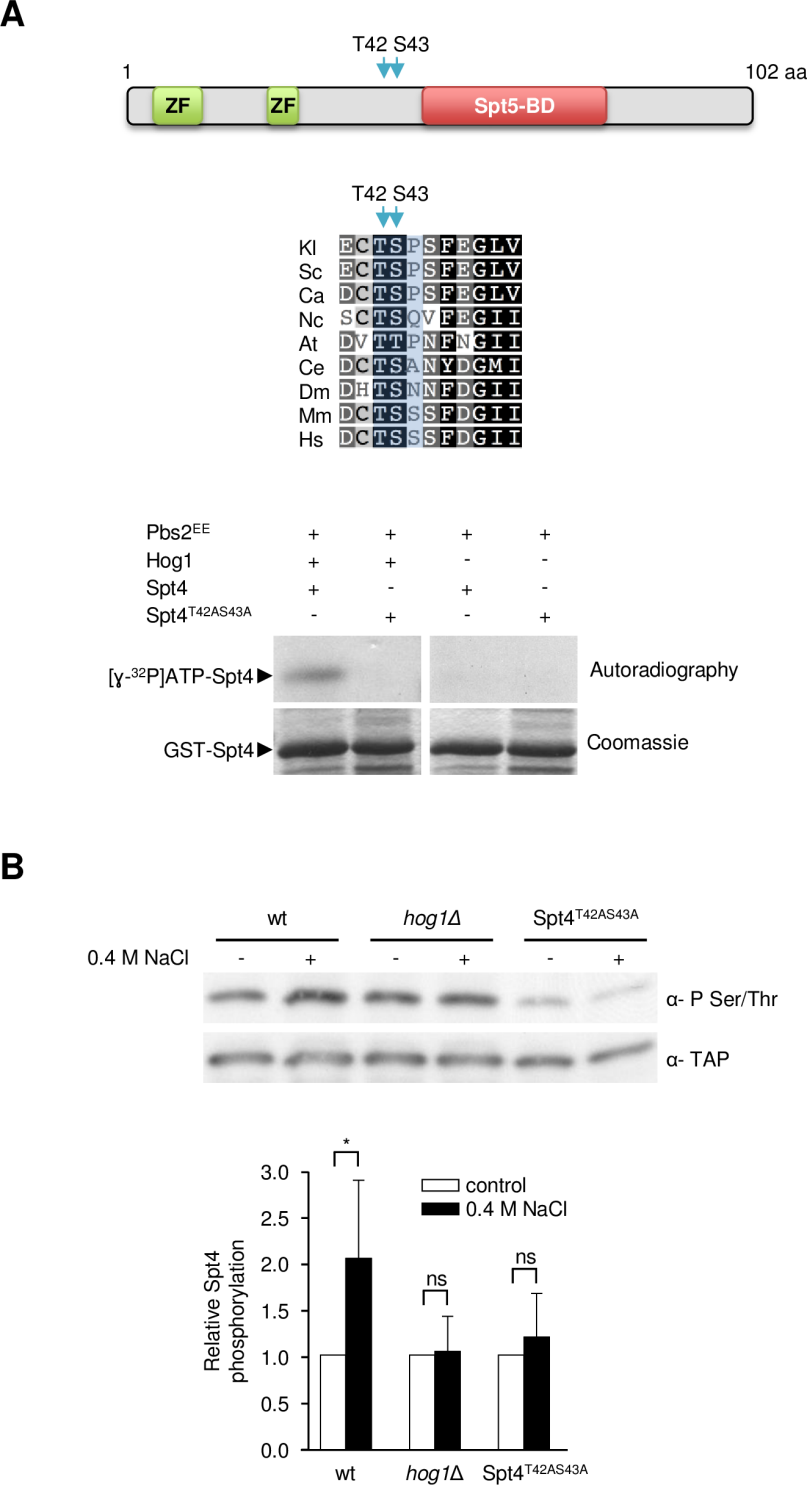
Spt4 is directly phosphorylated by Hog1. **(A)** Hog1 phosphorylates Spt4 at Thr42 and Ser43 *in vitro*. Upper panel: Schematic representation of the domain structure of Spt4 is shown. ZF, zinc finger domain; Spt5-BD, Spt5-binding domain. The arrows indicate the predicted sites of Hog1-dependent phosphorylation. Middle panel: Alignment of the relevant amino acid sequences of representative Spt4 homologs. Amino acid residues corresponding to the Hog1-dependent phosphorylation sites are highlighted in blue. Sc, *Saccharomyces cerevisiae*; Kl, *Kluyveromices lactis*; Ca, *Candida albicans*; Nc, *Neurospora crassa*; At, *Arabidopsis thaliana*; Ce, *Caenorhabditis elegans*; Dm, *Drosophila melanogaster*; Mm, *Mus musculus*; Hs, *Homo sapiens*. Lower panel: *E*. *coli* GST-purified Spt4 wild type and Spt4^T42AS43A^ mutant (harboring mutations that change Thr42 and Ser43 to Ala) versions were used in an *in vitro* Hog1 kinase assay. Proteins were resolved by SDS-PAGE and phosphorylated proteins were detected by autoradiography. The total protein level was detected by staining with Coomassie Blue. **(B)** Hog1 phosphorylates Spt4 at Thr42 and Ser43 *in vivo*. The indicated strains with TAP-tagged Spt4 were subjected or not to osmostress (0.4 M NaCl, 5 minutes). TAP-tagged Spt4 in protein extracts was then immunoprecipitated using rabbit IgG agarose and phosphorylated Spt4 was detected by immunoblotting using an anti-phospho Ser/Thr antibody (BD Transduction Laboratories). The mean ratio of phosphorylated versus total Spt4 ± SD of three different experiments, normalized to the value at t = 0, is shown. ns means p≥0.1; * means p< 0.1.

To elucidate the role of Hog1 in transcription elongation, we tested whether Hog1 directly phosphorylates any of the RNA Pol II transcription elongation factors. We systematically purified a subset of 40 TAP-tagged proteins involved in transcriptional elongation and subjected them to an *in vitro* phosphorylation assay with active Hog1 (S1 Fig). Of these proteins, we found 3 transcriptional-related factors (Spt6, Spt5 and Spt4) that were phosphorylated by Hog1 and followed up the phosphorylation event of the elongation factor Spt4. We focused on Spt4 since it has a unique putative phosphorylation consensus site for the SAPK and it was suggested to be involved in the osmostress response [21]. To validate this result, we used GST-tagged Spt4 purified from *Escherichia coli* in an *in vitro* kinase assay with Hog1 that was activated in the presence of a constitutively active MAPKK allele (Pbs2^EE^) [15]. As shown in Fig 1A, Spt4 was phosphorylated only when it was incubated with activated Hog1, indicating that it is a direct substrate for this SAPK. We next determined the sites of Spt4 phosphorylation by Hog1. Spt4 contains the sequence Thr42-Ser43-Pro44 that coincides with the putative S/TP MAPK consensus site and combined mutations of Thr42 (and the adjacent Ser43) to Ala resulted in total loss of phosphorylation by Hog1 (Fig 1A). It is worth noting that these sites in Spt4 are extremely conserved throughout evolution, although in some cases the proline is not conserved (Fig 1A).

To assess Spt4 phosphorylation *in vivo*, endogenous TAP-tagged Spt4 was immunopreciptated from wild type and *hog1Δ* mutant cells that were subjected or not to osmostress, and Spt4 phosphorylation was analyzed by Western blotting using anti-pSer/Thr antibodies as described in [35]. As shown in Fig 1B, Spt4 phosphorylation increased upon stress and this phosphorylation was dependent on Hog1 since it did not increase in *hog1Δ* mutant cells. This Spt4 phosphorylation was specific to the Hog1 phosphorylation sites (Thr42 and Ser43) since it was abolished in cells carrying the TAP-tagged mutant allele Spt4^T42AS43A^ (Fig 1B). These data indicated that Spt4 is phosphorylated in response to stress by Hog1. It is worth noting that the Spt4 is already phosphorylated in the absence of stress, suggesting that other non-stress kinases might act on these sites in basal conditions. Of note, Spt4 might also be phosphorylated at other sites than Thr42 and Ser43 in basal conditions, given that there is still signal with the antibody anti-P Ser/Thr in the Spt4^T42AS43A^ mutant.

### Phosphorylation of Spt4 by the Hog1 SAPK is required for the transcriptional response upon osmostress

**Fig 2:**
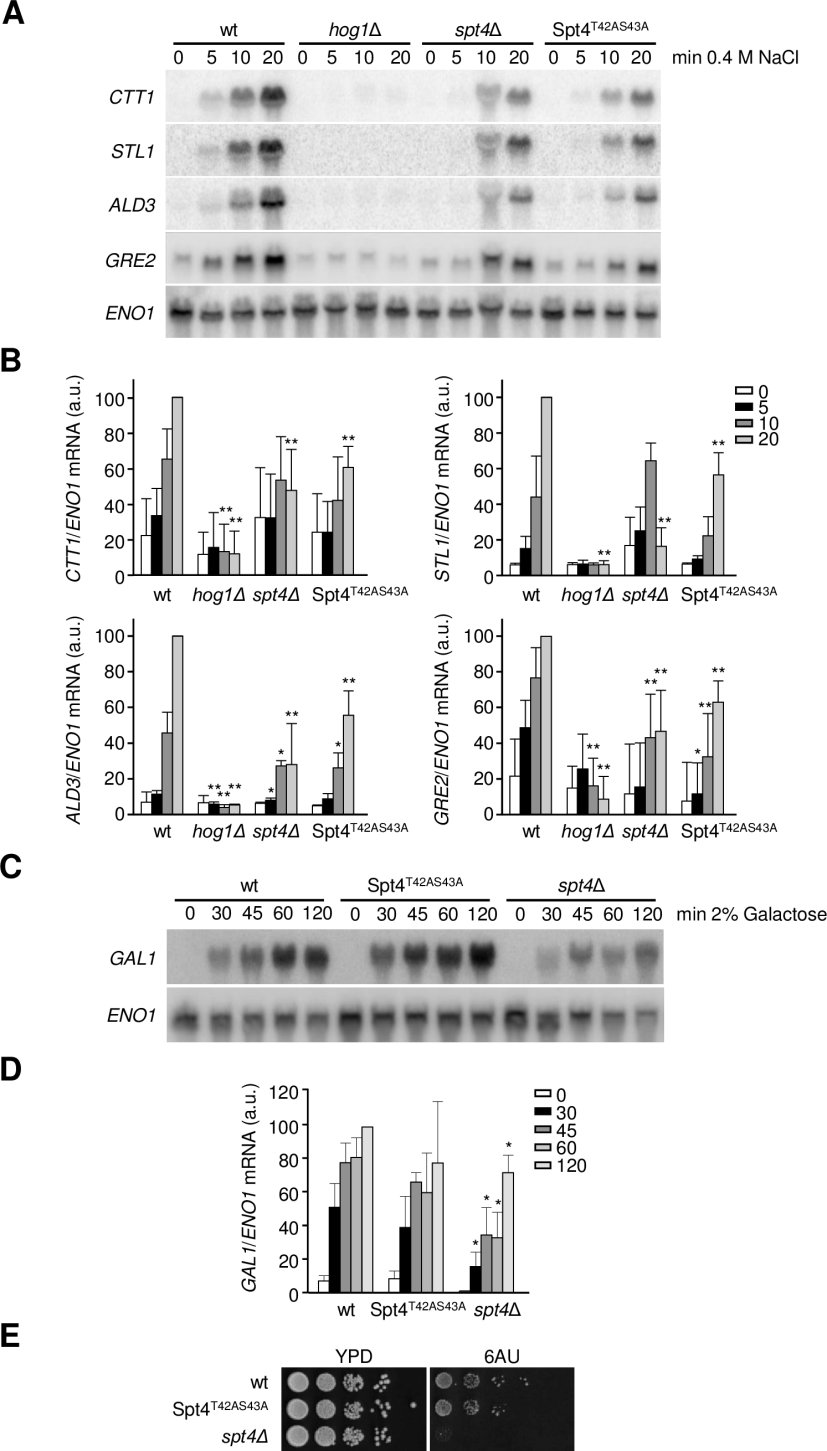
Spt4 phosphorylation sites are required for osmostress gene expression. **(A)** The indicated strains were subjected to osmostress (0.4 M NaCl) for the indicated times. Total RNA was analyzed by Northern blotting with radiolabeled probes specific for *CTT1*, *STL1*, *ALD3*, *GRE2* (osmostress genes) and *ENO1* (as a loading control) mRNA. Expression was measured relative to the loading control of each mRNA. **(B)** Quantification of relative expression levels of *CTT1*, *STL1*, *ALD3* and *GRE2*. Results are expressed as the percentage of the ratio with *ENO1* expression, normalized to the maximum level of expression in the wild type which is shown as 100%. Data of three biological replicates were used and means ± SD were calculated. t-test was calculated between each time point of the mutants and the wild type strains. Only significant differences are indicated; *, p<0.1; **, p<0.05. **(C)** The phosphorylation sites of Spt4 are not required for expression of the *GAL1* gene. Wild type and the indicated mutant strains were shifted to 2% galactose medium for the indicated times. Total RNA was analyzed by Northern blotting with radiolabeled probes specific for *GAL1* and *ENO1* (as a loading control). **(D)** Quantification of relative expression levels of *GAL1*. Results are expressed as in (B). Data of two biological replicates were used and means ± SD were calculated. t-test was calculated between each time point of the mutants and the wild type strains. Only significant differences are indicated; *, p<0.1. **(E)** The Spt4^T42AS43A^ mutant does not display a general elongation defect. Wild type (wt) and the indicated *spt4* mutant strains were grown to mid-log exponential phase and 10-fold serial dilutions were spotted on YPD and synthetic complete media containing 0.1 mg/ml 6-azauracil (6-AU). Growth at 30°C was scored after 4 days.

Cells lacking *SPT4* have reduced expression of osmostress-responsive genes [21]. To further study the functional relevance of Spt4 phosphorylation by Hog1 for the osmostress response, we followed the transcriptional response of cells expressing the non-phosphorylatable allele Spt4^T42AS43A^ upon osmostress. This mutant displayed reduced expression of representative stress-responsive genes (*STL1*, *CTT1*, *ALD3* or *GRE2*) versus wild type cells, but similar expression to that of *spt4Δ* cells (Fig 2A and 2B), indicating that phosphorylation of Spt4 on these specific residues is relevant for the regulation of osmostress-dependent gene expression. It is worth noting that osmo-responsive gene expression of the untagged and TAP-tagged Spt4 strains was similar upon osmostress (S2A and S2B Fig). Moreover, although the protein levels of the Spt4^T42AS43A^ mutant were quite similar as compared to wild type Spt4 strain, Spt4 mutant expression was only slightly reduced (20%) (S2C Fig). The defect in transcription that was triggered by the non-phosphorylatable mutant of Spt4 was specific to stress, since, in contrast to the deletion of *SPT4* (*spt4Δ*), it did not affect the expression of *GAL* genes upon galactose addition (Fig 2C and 2D). Of note, cells carrying the deletion of *SPT4* have been shown to be sensitive to 6-azauracil (6-AU), which is frequently used as a tool to study the genetics of transcript elongation [24]. In contrast to *spt4Δ* cells, cells expressing the non-phosphorylatable Spt4 mutant could grow in the presence of 6-AU to the same extent as wild type cells (Fig 2E), which indicated the functionality of the Spt4^T42AS43A^ allele. These data confirm that the phosphorylation of Spt4 by Hog1 is relevant in the control of stress-responsive genes.

**Fig 3:**
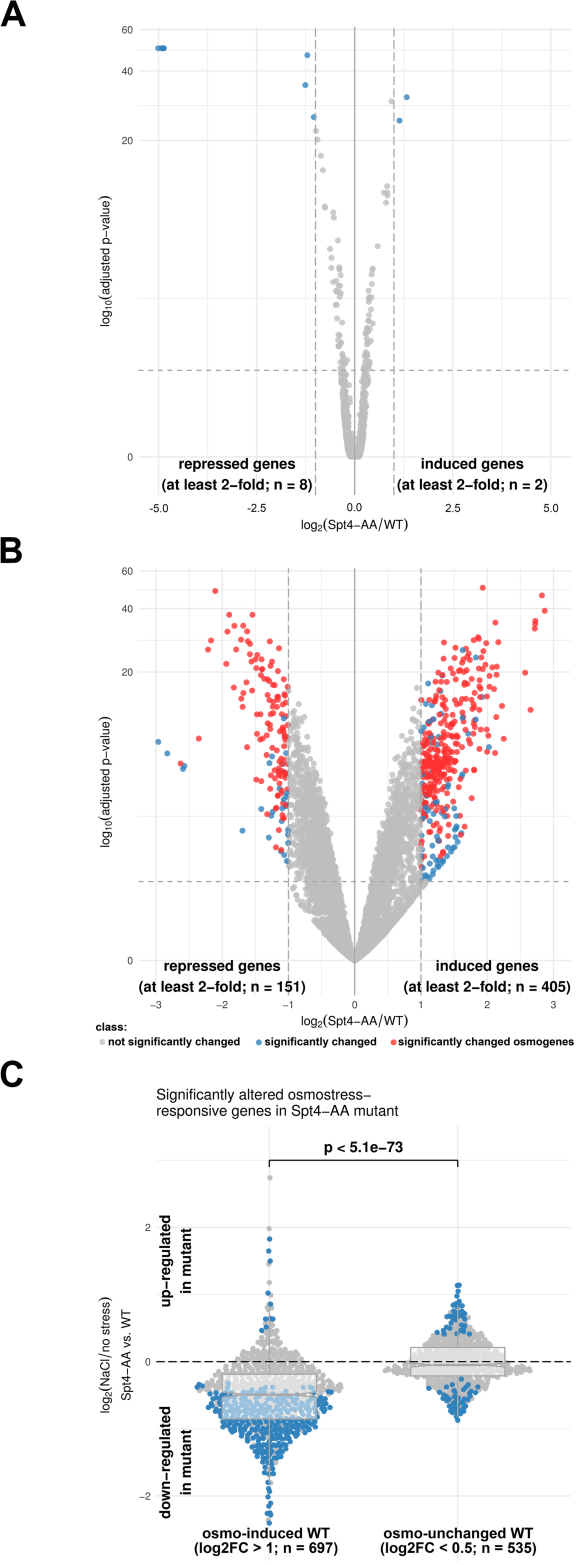
The Spt4^T42AS43A^ mutant is deficient in the induction of stress-responsive genes. **(A)** Volcano plot showing significantly altered gene expression of Spt4^T42AS43A^ (Spt4-AA) mutant versus wild type (WT) strains in non-stressed conditions. n indicated the number of genes significantly altered in the Spt4^T42AS43A^ mutant compared to wild type. **(B)** Volcano plot showing significantly altered gene expression of Spt4^T42AS43A^ (Spt4-AA) mutant versus wild type (WT) strains upon stress (0.4 M NaCl, 15 min). n indicated the number of genes significantly altered in the Spt4^T42AS43A^ mutant compared to wild type. Known osmo-responsive genes are shown in red dots. **(C)** Differences in the transcriptional stress-activated and stress-unchanged response of wt and Spt4^T42AS43A^ mutant cells. Horizontal lines in the boxplots indicate the median, dashed grey lines indicate a relative absolute fold change of 1. The indicated p-value is based on a Wilcoxon-test. Blue dots mark genes with significantly altered expression (A and B: |log2FC| > 2, FDR < 0.05, C: FDR < 0.05).

To further characterize the effect of the Hog1-phosphorylation sites in Spt4 on osmostress-induced gene transcription, we characterized the genome-wide gene expression in response to osmostress by performing RNA-seq in wild type and Spt4^T42AS43A^ cells. Under stress conditions (0.4M NaCl, 15 min.), expression profiles of 1507 genes were found to be significantly altered by at least 2-fold (FDR< 0.05) in the wild type strain (S3 Fig). Under non-stressed conditions the wild type and Spt4^T42AS43A^ mutant displayed a very similar gene expression pattern, with only a few genes significantly altered in the mutant versus the wild type (FDR < 0.05 and at least 2-fold difference) (Fig 3A). These results indicated that, in contrast to the deletion of *SPT4* [34], the unphosphorylatable Spt4 mutant had only minor effect on the overall transcriptional pattern in basal conditions. However, upon osmostress, expression of 556 genes was significantly altered in the Spt4^T42AS43A^ mutant cells compared to wild type (FDR < 0.05 and at least 2-fold difference) (Fig 3B). When the osmostress-induced transcriptional response was further analyzed, we observed that almost 50% (335 genes) genes that are normally osmo-induced in wild type cells exhibited a significantly smaller induction in Spt4^T42AS43A^ mutant cells (Fig 3C). Notably, osmo-unchanged genes (defined by log2 fold change significantly smaller than 0.5, FDR < 0.05) did not show a comparable systematic difference between wild type and Spt4^T42AS43A^ strains. These results led us to conclude that phosphorylation of Spt4 is required for proper activation of genes responsive to osmostress.

### Phosphorylation of Spt4 by the Hog1 SAPK is required for stress-induced transcription elongation

**Fig 4:**
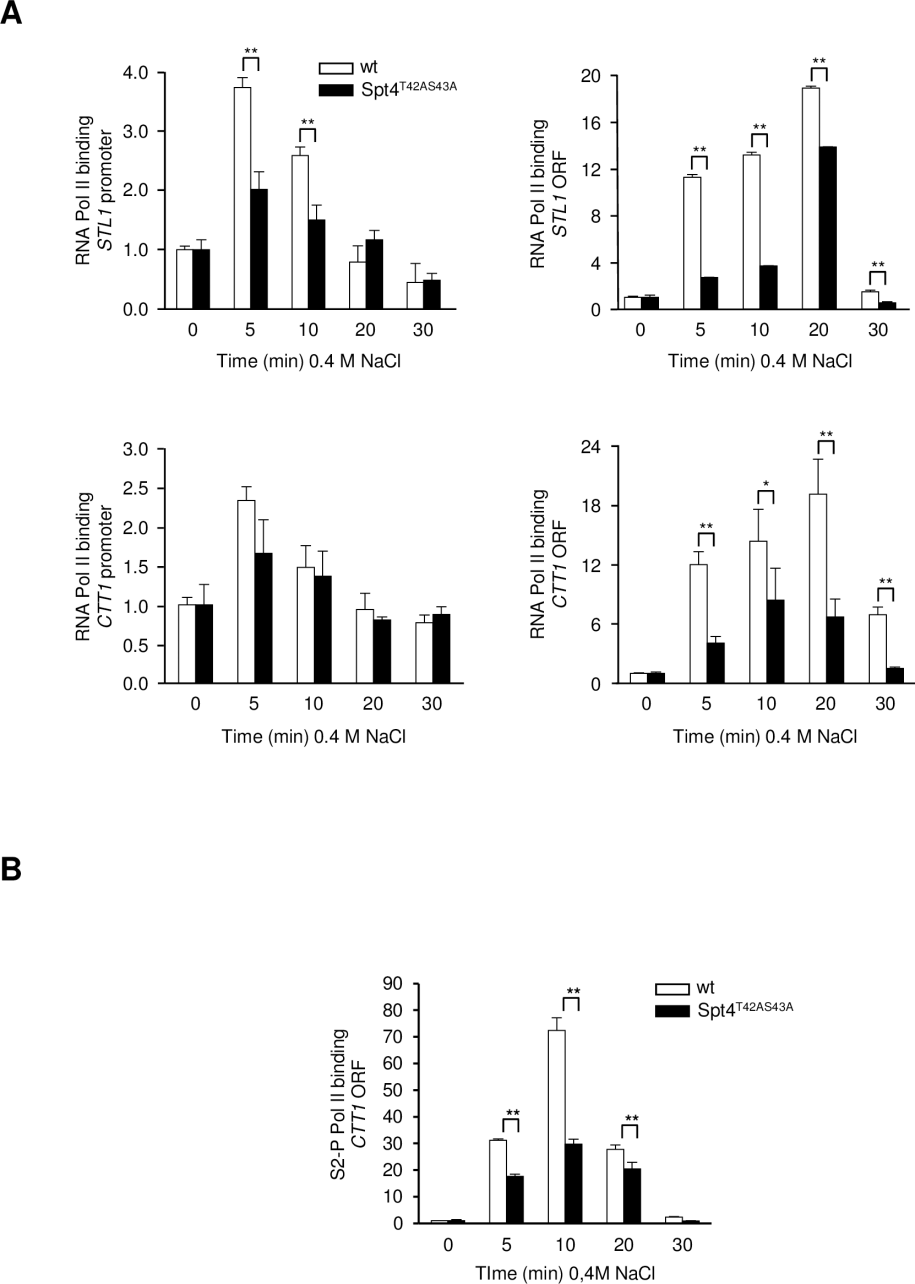
Binding of RNA Pol II to osmo-responsive genes is impaired in the Spt4^T42AS43A^ mutant. **(A)** Association of RNA Pol II at stress-dependent loci was monitored by ChIP in wild type (wt; white bars) and Spt4^T42AS43A^ (black bars) strains upon osmostress (0.4 M NaCl at the indicated times). Chromatin-bound RNA Pol II was immunoprecipitated using the monoclonal anti-Rpb1 antibody (8WG16, Covance). The precipitate was analyzed by real-time PCR using primers specific for the *CTT1* or the *STL1* promoter (left panel) and ORF (right panel) regions. **(B)** Association of Ser2-P RNA Pol II at stress-dependent *CTT1* locus was monitored by ChIP as in A, using rabbit anti-phospho RNA Polymerase II (S2) antibody (Bethyl Laboratories). Data are the means ± SD of three technical replicates from a representative experiment and were normalized to a telomeric region used as an internal control. t-test was calculated between each time point of the mutants and the wild type strains. Only significant differences are indicated; *, p<0.1; **, p<0.05.

We then assessed the association of RNA Pol II (Rpb1) with representative stress-responsive genes in wild type and Spt4^T42AS43A^ cells using ChIP analysis (Fig 4A). As expected, RNA Pol II was recruited to the promoters and ORFs of *STL1* and *CTT1* only in response to osmostress. Cells carrying the Spt4^T42AS43A^ mutation showed reduced binding of RNA Pol II to *STL1* and *CTT1* in response to stress compared to wild type, and the effects were particularly striking for the coding regions of those genes. These results are consistent with the fact that *CTT1* and *STL1* gene expression is reduced in response to stress in the non-phosphorylatable mutant (Fig 2A). Of note, ChIP assays using a specific antibody that recognizes RNA Pol II Ser2-CTD showed that association of RNA Pol II phosphorylated at Ser2 with the stress-dependent *CTT1* coding region was reduced in cells carrying the Spt4^T42AS43A^ mutation compared to wild type (Fig 4B).

**Fig 5:**
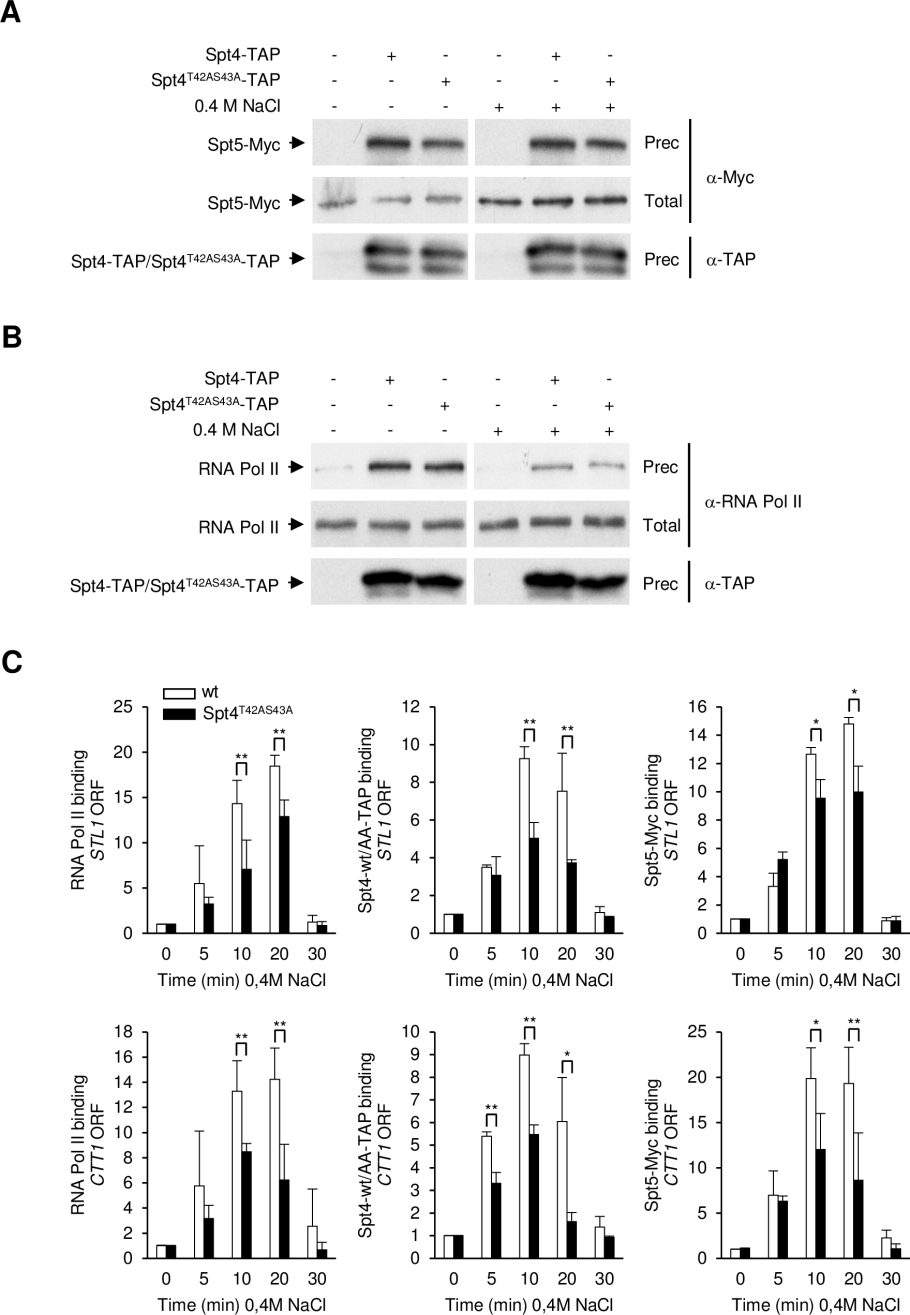
**(A, B)** *In vivo* binding of Spt4 with Spt5 and RNA Pol II. Spt4-TAP, Spt4^T42AS43A^-TAP and Spt5-Myc were expressed from the endogenous locus. Samples were taken before (-) or 10 min after (+) the addition of 0.4 M NaCl. Spt4-TAP was immunoprecipitated with antibodies against TAP. (A) Spt4-/Spt4^T42AS43A^-TAP (bottom) and Spt5-Myc (top) proteins were detected by Western blotting with anti-TAP and anti-Myc antibodies, respectively. Total, 2.5% of total input protein (middle); Prec, precipitated proteins. (B) Spt4-/Spt4^T42AS43A^-TAP (bottom) and RNA Pol II (top) proteins were detected by Western blotting with anti-TAP and anti-Rpb1 antibodies, respectively. Total, 2.5% of total input protein (middle); Prec, precipitated proteins. **(C)** Binding of Spt4 and Spt5 to osmo-responsive genes is impaired in the Spt4^T42AS43A^ mutant. Association of RNA Pol II (left panels), Spt4-TAP (middle panels) and Spt5-Myc (right panels) at stress-dependent loci was monitored by ChIP in Spt5-Myc Spt4-TAP tagged (wt; white bars) or Spt5-Myc Spt4^T42AS43A^-TAP tagged (black bars) strains upon osmostress (0.4 M NaCl at the indicated times). The precipitate was analyzed by real-time PCR using primers specific for the *STL1* (upper panels) or the *CTT1* (lower panels) ORF regions. Data are the means ± SD of three experimental replicates and were normalized to a telomeric region used as an internal control. t-test was calculated between each time point of the mutant strains and the wt strain. Only significant differences are indicated; *, p<0.1; **, p<0.05.

The complex of Spt5-Spt4 is required for efficient transcription elongation by RNA Pol II [24,25]. The sites in Spt4 that are phosphorylated by Hog1 are located close to the Spt5-binding domain (Spt5 NGN) and, when modeled, they appear to be accessible. Thus, it is formally possible that the phosphorylation of Spt4 resulted in a change in the affinity of the binding of Spt4 with Spt5, or with the indirect binding of Spt4 with RNA Pol II to stimulate transcription processivity. This possibility was addressed by testing whether Spt4 wild type or the non-phosphorylatable mutant were differently immunoprecipitated with Spt5 (Fig 5A) or RNA Pol II (Fig 5B) in response to osmostress. Yeast cells expressing TAP-tagged Spt4 or Spt4^T42AS43A^ and Myc-tagged Spt5, each expressed from their genomic locus, were subjected to osmostress and Spt4 was immunoprecipitated using specific antibodies against TAP. As shown in Fig 5A and 5B, Spt4 wild type and its non-phosphorylatable version coprecipitated Spt5 and RNA Pol II upon stress to the same extent, indicating that Spt4-Spt5 and Spt4-RNA Pol II interactions are not affected by Hog1-phosphorylation of Spt4. Next, we assessed Spt4 and Spt5 binding to osmo-responsive genes in wild type and Spt4^T42AS43A^ cells by ChIP assays. As expected, Spt4 and Spt5 were recruited to the coding regions of *STL1* and *CTT1* only in response to osmostress. Association of Spt4 and Spt5 to osmo-responsive coding regions was impaired in the non-phosphorylatable Spt4 mutant when compared to wild type (Fig 5C). In addition, we followed in the same cultures the association of RNA Pol II and found that it was reduced similarly (Fig 5C). Therefore, it is conceivable that the association of Spt4 and Spt5 to chromatin is reduced in the non-phosphorylatable Spt4 mutant due to a decrease in RNA Pol II association.

**Fig 6:**
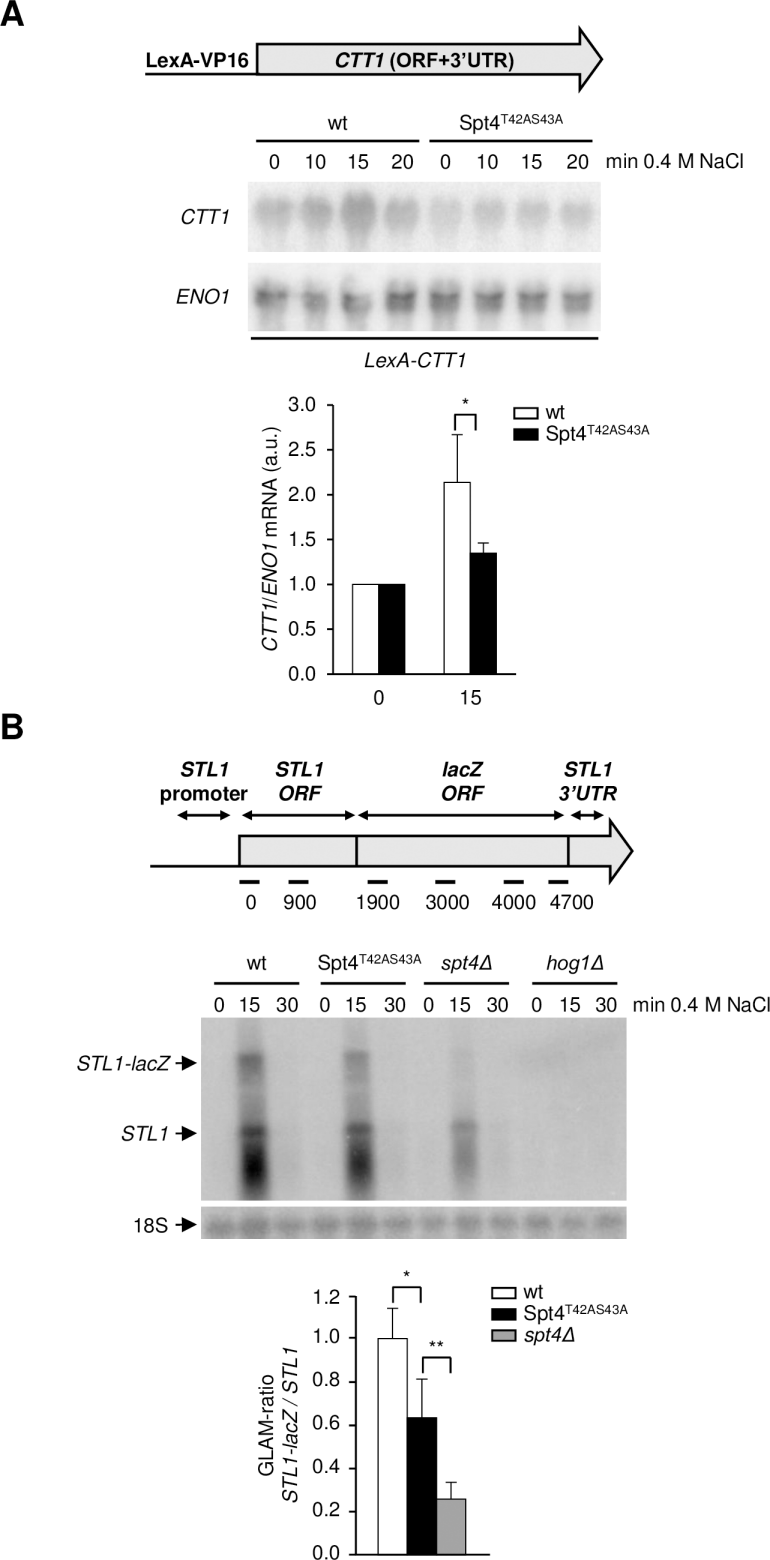
Hog1-dependent Spt4 phosphorylation is required for stress-dependent RNA levels. **(A)** The Spt4^T42AS43A^ mutant showed a decrease in *CTT1* mRNA expression versus wild type (wt) when induced by the LexA-Vp16 activator. The indicated strains lacking endogenous *CTT1* were transformed with a vector carrying *CTT1* under the control of the LexA promoter and with a plasmid containing the LexA binding domain fused to the VP16 transcriptional activator. Cells were subjected to osmostress (0.4 M NaCl) for the indicated times and total RNA was extracted and analyzed by Northern blotting as in Fig 2A. Lower panel: Quantification of relative expression levels of *CTT1*. *ENO1* was used as a loading control. Fold induction is expressed as the ratio with *ENO1* expression, normalized to non-stress condition. Data of three biological replicates were used and means ± SD were calculated. t-test was calculated between each time point of the mutant strains and the wt strain. *, p<0.1. **(B)** Upper panel: Schematic diagram of an osmostress-dependent long transcription unit with the distribution of the amplicons and probes utilized to measure RNA Pol II occupancy along *STL1*-*lacZ*. *STL1* promoter: 810 bp; *STL1* ORF: 1710 bp; *lacZ* ORF: 3100 bp; *STL1* 3’UTR: 436 bp. Middle panel: The Spt4^T42AS43A^ mutant exhibited lower ability to express a long mRNA versus other strains. The indicated strains were transformed with *STL1*-*lacZ* and expression of both the long *STL1*-*lacZ* and the short endogenous *STL1* transcription units upon stress (0.4 M NaCl, at the indicated times) was assessed by Northern blotting with a radiolabeled probe specific for *STL1*. A representative of three Northern experiments is shown. Lower panel: The quantification of expression in three biological replicates is shown. GLAM ratios were calculated by dividing the amount of *STL1*-*lacZ* mRNA by the amount of endogenous *STL1* mRNAs after 15 minutes of osmostress. Values correspond to the mean and the standard deviation of three biological replicates normalized to the wild type. *p<0.05, **p<0.005 using Student’s t-test.

To analyze the role of Spt4 phosphorylation by Hog1 within the RNA Pol II elongation complex, we uncoupled the processes of transcription initiation and elongation in response to osmostress by driving the expression of an osmo-inducible gene with the LexA-VP16 activator. By this approach, the stress-dependent transcript levels can be attributed exclusively to an effect on elongation and not to changes in transcriptional initiation [21]. Specifically, we measured RNA levels of the *CTT1* osmo-responsive gene when their expression was driven by the stress-independent LexA promoter and the LexA-VP16 activator (Fig 6A). In this system, in which initiation of transcription is independent from stress and Hog1, but the osmo-inducible coding region is regulated by stress in a Hog1-dependent manner, the amount of *CTT1* mRNA still increases in response to stress. This induction is impaired in the Spt4^T42AS43A^ mutant, suggesting that these phosphorylation events are required for a proper RNA levels upon osmostress.

### Spt4 phosphorylation by the Hog1 SAPK regulates RNA Pol II processivity in stress-responsive genes

Mutants with impaired transcription elongation display lower efficiency in the gene expression of long compared to short transcription units [36]. Gene Length Accumulation of mRNA (GLAM)-ratios was previously used as an indirect estimation of RNA Pol II elongation. For instance, the *spt4Δ* mutant shows low GLAM-ratios [37]. To gain insight into the putative defects in stress-dependent transcription elongation of the non-phosphorylatable Spt4 mutant, we engineered an osmostress-inducible long transcription unit by fusing the *STL1* gene (1710 bp) with *lacZ* (4810 bp) (Fig 6B). This unit was expressed in wild type, Spt4^T42AS43A^ or *spt4* mutant strains and the GLAM ratios were determined by dividing *STL1* expression of the long *STL1*-*lacZ* transcription unit versus the short endogenous *STL1* transcription unit upon osmostress (Fig 6B). *spt4* deleted cells showed GLAM values below 0.5 in response to stress as expected from a mutant with defects in RNA Pol II elongation. Notably, the levels of the full-length mRNAs transcribed from *STL1*-*lacZ* upon stress were significantly reduced in Spt4^T42AS43A^ as compared to the short mRNA from *STL1*, suggesting that phosphorylation of Spt4 by Hog1 upon stress influences gene expression in a gene length-dependent way and is therefore important for transcription elongation.

**Fig 7:**
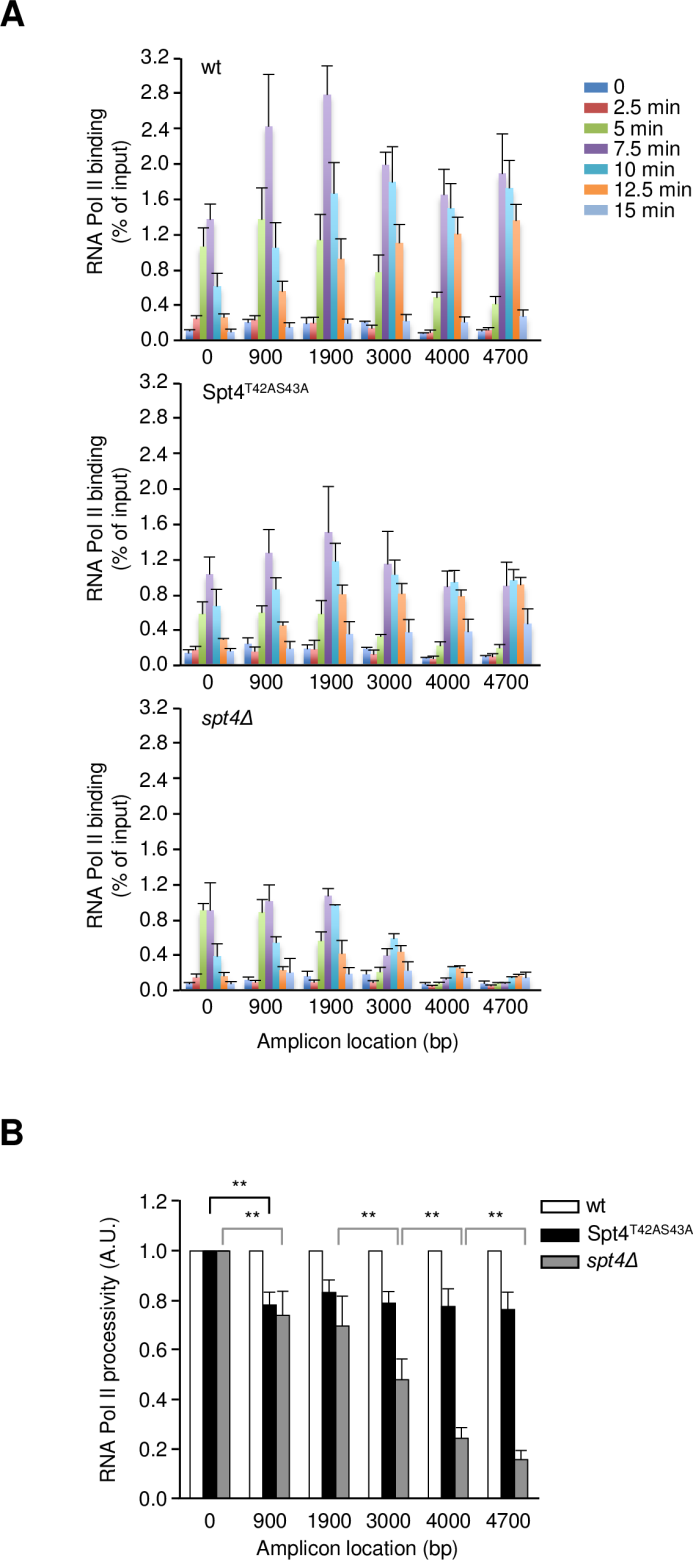
Hog1-dependent Spt4 phosphorylation is required for elongation in the transcription of osmo-responsive genes. **(A)** Association of RNA Pol II at *STL1-lacZ* was monitored by ChIP using the monoclonal anti-Rpb3 antibody (ab81859; Abcam) in wild type (wt), Spt4^T42AS43A^ and *spt4Δ* cells upon osmostress (0.4 M NaCl at intervals of 2.5 minutes for a total of 15 minutes). Values correspond to the mean of % immunoprecipitation (IP) / input and the standard deviation of three biological replicates. The first two amplicons are represented as 50% of the original value, taking into account that these primers detect both *STL1-lacZ* and endogenous *STL1*. **(B)** RNA Pol II processivity of Spt4^T42AS43A^ and *spt4Δ*, relative to the wild type, was calculated from the values obtained in (A). The bars represent the sum of the RNA Pol II amounts in all the time-points measured on each amplicon, normalized to the first amplicon and to the wild type. Values correspond to the mean and the standard deviation of three biological replicates. **p<0.01 using Student’s t-test.

We then aimed to assess whether Spt4 phosphorylation by Hog1 changed the distribution of RNA Pol II along the transcribed *STL1*-*lacZ* in response to stress. For this purpose, the profile of RNA Pol II occupancy along *STL1*-*lacZ* was analyzed using anti-Rpb3 ChIP in wild type, Spt4^T42AS43A^ and *spt4* mutant strains (Fig 7A and S4 Fig). In all three tested strains RNA Pol II associated with the 0 amplicon (starting of the transcript region) upon osmostress and this association reached a maximum at 7.5 minutes after induction. From this time-point onwards, the recruitment of polymerase decreased until it approximated background levels after 15 minutes. RNA Pol II occupancy along the other amplicons along *STL1*-*lacZ* increased proportionally over time in wild type cells, following the propagation of the transcriptional wave. *spt4*Δ showed significant lower levels of RNA Pol II binding along the transcription unit (except for amplicon 0) compared to wild type, as expected from a mutant that affects RNA Pol II processivity [32]. Similarly, Spt4^T42AS43A^ also displayed reduced levels of RNA Pol II binding along *STL1*-*lacZ* compared to wild type (reduction of ~50% from amplicon 900 to the end of the transcript). We then assessed the RNA Pol II processivity of the two mutants relative to wild type. In the Spt4^T42AS43A^ mutant, a defect in processivity was detected at the 5’ end of the gene body, whereas in *spt4Δ* a progressive RNA Pol II drop-off was observed throughout the whole transcription unit (Fig 7B).

### Spt4 phosphorylation by the Hog1 SAPK is important for cell growth upon osmostress

**Fig 8:**
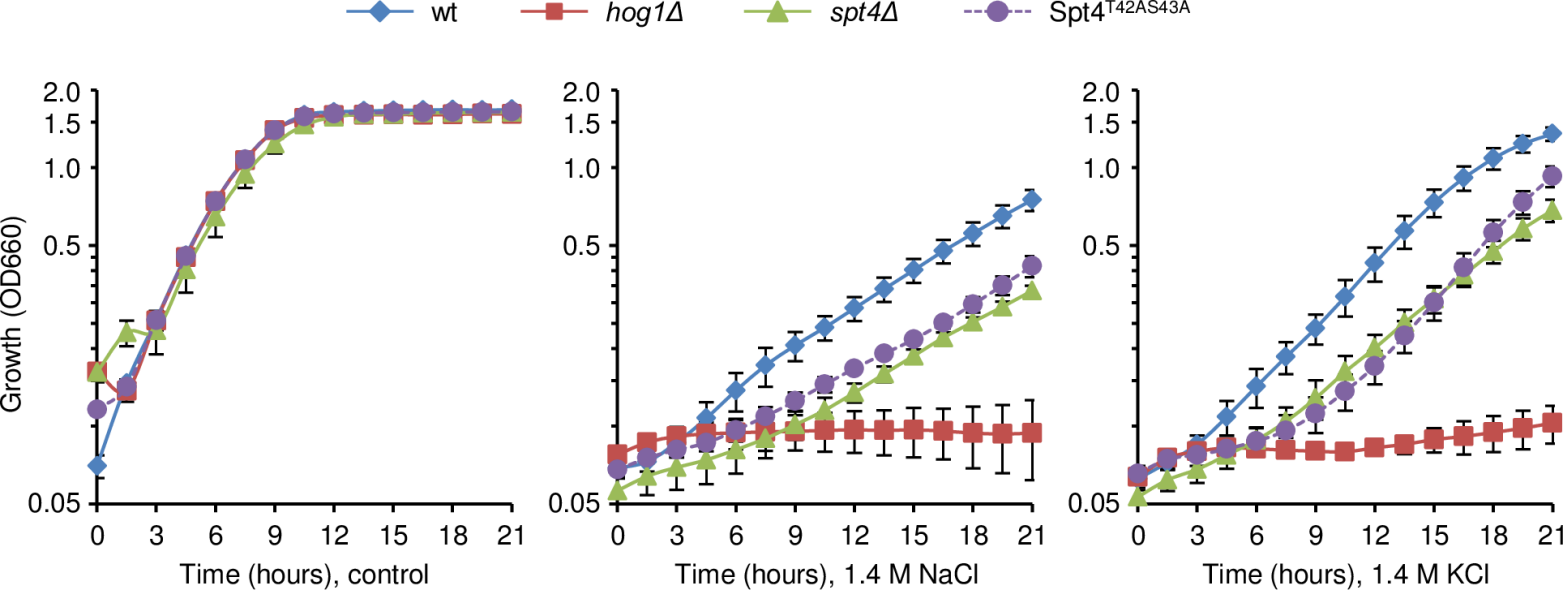
Spt4 phosphorylation by Hog1 is required for cell growth in the presence of osmostress. Wild type Spt4 (wt), *hog1Δ* and the indicated *spt4* mutant strains were grown to mid-log exponential phase in YPD and then diluted 1:10 in YPD, 1.4 M NaCl or 1.4 M KCl before starting the assay. Growth curves over 21 hours were analyzed using a Synergy H1 Hybrid Reader (BioTek). Data are the means ± SD of three technical replicates from a representative experiment. Growth curves are represented on a semi-log plot.

The role of transcription is not essential for the short-term adaptation, but it is crucial for long term adaptation and for protection against future stress (reviewed in [5]). Indeed, mutants that display impaired transcription at mild osmolarities display growth defects only under severe osmolarities. Thus, to assess the relevance of Spt4 phosphorylation and its control of transcription elongation for cell growth upon osmostress, we monitored cellular growth in the presence of high osmolarity (1.4 M NaCl or KCl). Cells lacking *HOG1* or *SPT4* show compromised cell adaptation to high osmolarity [21]. We therefore monitored the sensitivity of the Spt4^T42AS43A^ mutant to high osmolarity. The non-phosphorylatable Spt4 mutant did not show growth differences compared to wild type on YPD in the absence of osmostress. In clear contrast, the growth of the Spt4^T42AS43A^ cells was more sensitive to high osmolarities (1.4 M NaCl or KCl) than that of the wild type strain. Of note, Spt4^T42AS43A^ cells were as sensitive as *spt4Δ* mutant cells in this respect (Fig 8). Thus, alanine substitutions in phosphorylable residues of Spt4 confer similar osmostress sensitivity as deletion of *SPT4*, consistent with Hog1 phosphorylation of these residues being important for cell growth upon osmostress.

## Discussion

The Hog1 SAPK has a pivotal role in regulating distinct steps in the gene expression cycle in response to osmostress. Although the mechanisms by which Hog1 triggers stress-dependent transcriptional initiation are well-described (reviewed in [5,6]), those by which Hog1 contributes to the regulation of elongation remained to be explored. Transcription elongation by RNA Pol II is a dynamic and tightly regulated step that is facilitated by several transcription elongation accessory factors as well as chromatin remodelers (reviewed in [22,23]). We previously reported that Hog1 interacts with components of the RNA Pol II transcript elongation complex such as Spt4, Paf1, Dst1 or Thp1 and recruits the RSC chromatin remodeler to stress-responsive genes [4,12]. These elongation factors and chromatin remodelers are important for transcriptional activation in response to osmostress and for cell ability to grow under such conditions [21]. Here, we showed that the stress-activated Hog1 directly phosphorylates the Spt4 elongation factor to regulate the activity of RNA Pol II in response to osmostress.

Hog1 serves to bypass the general down-regulation of gene expression that occurs upon osmostress by targeting RNA Pol II and inducing chromatin remodeling at stress-responsive loci [4]. Here, we demonstrated that this signaling kinase also targets an elongation factor to govern the transcription of stress-responsive genes. Several evidences have pointed out the ability of *spt4* deletion to affect gene expression selectively rather than affect general transcription. For instance, RNA-seq analyses indicated that only a small fraction of the yeast transcriptome is affected in the *spt4* mutant [34]. It has also been reported that Spt4 is specially required for the expression of long genes [32] and for transcribing genes with high GC content [33]. Here, we presented evidences that Spt4 is also required for stress-responsive gene expression. The absence of phosphorylation of Spt4 by Hog1 does not result in major effects on gene expression compared to wild-type under non-stress conditions; however, the expression of stress-responsive genes is clearly impaired. Notably, many genes whose expression is compromised in the Spt4^T42AS43A^ mutant are also Hog1-dependent genes, which indicates a specific regulation of Spt4 activity by phosphorylation by Hog1. Although it is formally possible that the Thr42 and Ser43 amino acid changes altered the Spt4 function, the Spt4^T42AS43A^ mutant specifically showed a defect in transcription in response to osmostress but not upon galactose addition. Moreover, in contrast with cells deficient for Spt4 activity, cells expressing the Spt4^T42AS43A^ mutant did not display cell growth defects in the presence of 6-AU. Of note, the site in Spt4 that is phosphorylated by Hog1 is completely conserved from yeast to mammals, suggesting that this phosphosite might have a physiologically relevant functional role across all eukaryotic cells.

Genome-wide localization studies showed that the Spt4 and Spt5 were hardly ever present at promoters, but that their association with gene loci were strongly increased downstream of transcription start sites, which largely mirrored the distribution of RNA Pol II [38,39]. This finding suggested that Spt5/Spt4 binding to genes may serve to promote the initiation-elongation transition by sterically enforcing the nucleic acid arrangement and preventing RNA release and reassociation of DNA strands [29]. Also, it has been described that there is physical and genetic interaction between the osmostress initiation transcription factor Hot1 and Spt5/Spt4 [40]. Indeed, the presence of RNA Pol II at osmo-responsive promoters was slightly reduced in the unphosphorylatable Spt4 mutant, suggesting that phosphorylation of Spt4 by Hog1 might also be important for this transition and a potential cause for the changes observed during transcription elongation. The defect of RNA Pol II processivity across the *STL1*-lacZ fusion in the Spt4^T42AS43A^ mutant was limited to the beginning of the transcript compared to the *spt4Δ*, which showed a defect across all the transcript. This suggests that the phosphorylation of Spt4 by Hog1 upon osmostress modulated mainly early transcriptional elongation. Of note, it was shown that the role of Spt4/5 has also been involved in the transition of initiation to elongation, where they compete with the initiation factor TFIIE for its binding to RNA Pol II to stimulate processivity [41]. Thus, it might be that stress-dependent Spt4 phosphorylation stimulates RNA Pol II processivity in an early step of transcriptional elongation.

Crystal structure, modelling and *in vivo* crosslinking studies of the conserved complex of Spt4 with its partner Spt5 showed that it binds to the coiled domain of RNA Pol II and encloses the DNA template to promote processivity [28,29,31,42]. However, by co-imunoprecipitation assay, we did not detect a change in the affinity of Spt4 for Spt5 or RNA Pol II when it cannot be phosphorylated upon stress. Of note, Spt5 is also targeted by Hog1 *in vitro* and a mutant strain carrying Spt5 mutations in the putative Ser and Thr MAPK consensus sites to Ala showed compromised cell growth to high osmolarity. However, we did not observe any additivity on osmostress sensitivity when we combined it with the Spt4 mutation. Thus, we focused in the regulation of Spt4 to understand the effect of the phosphorylation in elongation. Spt4 does not directly contact RNA polymerase, but it stabilizes RNA Pol II/template complexes by binding to the template externally to the transcription bubble [28,29]. It is known that *spt4* and *spt5* mutations affect the ability of elongating RNA Pol II to traverse the entire length of a gene [32]. The association of Hog1 with coding regions increases RNA Pol II density at osmo-responsive coding regions [21]. Thus, it is reasonable to assume that cells have evolved mechanisms, such as the direct regulation of Spt4, to ensure proper polymerase processivity through those genes that should be activated rapidly. On the other hand, Spt4 phosphorylation by Hog1 may also function at a step distinct from elongation such as stability of target RNAs or RNA export.

Although the Ras/PKA signaling pathway is somehow involved in modulation of the Spt5/Spt4 complex [43], the regulation of this elongation complex by external cues has not been reported. The fact that the direct phosphorylation of Spt4 by a signaling kinase facilitates RNA Pol II processivity suggests that elongation can be modulated depending on external cues to increase transcription efficiency and maximize cell fitness.

## Materials and methods

### Yeast strains

All strains are based in the BY4741 (*MAT***a** *his3-Δ1 leu2-Δ0 met15-Δ0 ura3-Δ0)* genetic background. Genetic deletions and taggings were performed on the corresponding native genomic loci and under the control of their own promoters, using a long flanking homology PCR-based approach [44,45].

To genomically introduce Thr42Ala and Ser43Ala point mutations into Spt4 (YAS13 strain), a *Spt4(T42A S43A)-TAP*::*kanMX4* cassette was generated by PCR in a two-step reaction. The first PCR product was amplified using oligos OAS60 (AGGGCTCGAAGGATGTACTG) and OAS71 (CCCTCGAAAGAAGGAGCA GCACATTCCATT) with genomic DNA from the BY4741 *SPT4-TAP*::*kanMX4* strain (YAS3) as a template, yielding a mutagenic PCR product. This product was used as a primer in the second step together with oligo OAS76 (AGGGTCATAGTAGTCAAAGG) using YAS3 genomic DNA again as a template. The resulting *Spt4(T42A S43A)-TAP*::*kanMX4* PCR product was used for BY4741transformation; G418-resistant colonies were confirmed by PCR with oligos OAS60/OAS76, by sequencing and by Western blotting with the peroxidase anti-peroxidase (PAP) antibody (P1291, Sigma).

The strains BY4741 *SPT4-TAP*::*kanMX4* (YAS3), BY4741 *SPT4-TAP*::*kanMX4 hog1*::*URA3* (YAS7), *spt4*::*kanMX4* (YAS17), *hog1*::*URA3* (YCS40), *SPT4-TAP*::*kanMX4 stl1*::*LEU2* (YLS221), *SPT4-TAP*::*kanMX4 ctt1*::*LEU2* (YLS262), *Spt4(T42AS43A)-TAP*::*kanMX4 stl1*::*LEU2* (YLS222), *Spt4(T42AS43A)-TAP*::*kanMX4 ctt1*::*LEU2* (YLS264), *SPT5-9myc*::*hphNT1* (YSC29), *SPT4-TAP*::*kanMX4 SPT5-9myc*::*hphNT1* (YSC30) and *Spt4(T42AS43A)-TAP*::*kanMX4 SPT5-9myc*::*hphNT1* (YSC31) were made using standard procedures.

### Plasmids

Plasmids pGEX4T-Hog1 and pGEX4T-Pbs2^EE^ (*PBS2* with Ser514-Glu and Thr518-Glu mutations) were previously described [46]. *SPT4* was PCR amplified from BY4741 genomic DNA with the oligos OAS22 (GATCGGATCCTCTAGTGAAAGAGCCTGTAT) and OAS23 (GATCCTCGAGTTACTCAACTTGACTGCCATC), and was then *BamH*I/*XhoI* digested and cloned into pGEX-6P-1. *Spt4*^*T42AS43A*^ was amplified by PCR in a two-step reaction. The first PCR product was amplified from BY4741 genomic DNA with the oligos OAS40 (AATGGAATGTGCTGCTCCTTCTTTCGAGGG) and OAS23. The resulting mutagenized PCR product was used as a primer for the second step together with OAS22 and using BY4741 genomic DNA as a template. The final PCR product was digested with *BamH*I/*Xho*I and cloned into pGEX-6P-1. Both plasmids were confirmed by digestion and sequencing.

The *LexA-CTT1* construct, and the plasmid with the LexA binding domain fused to the VP16 transcriptional activator, have been described [21], and were used for transformation of the indicated yeast strains. For the GLAM assay, the *STL1* promoter and ORF (-810 to +1710 bp) were PCR amplified with the oligos OAS142 and OAS120 and cloned into pRS413 digested with *Xho*I*/Sal*I. The resulting plasmid was digested with *Sal*I*/Xma*I to clone the *lacZ* sequence (3100 bp) from pSCH212 [47] that had been amplified with the oligos OAS121 and OAS122. Finally, 430 bp from the *STL1* 3’ UTR were PCR amplified with the oligos OAS123 and OAS124 and cloned after *Xma*I*/Xba*I digestion. The plasmid was confirmed by sequencing.

### *In vitro* kinase assays

GST fusion proteins encoding Hog1, the constitutively active Pbs2^EE^ and the wild type or the mutant versions of Spt4, were expressed in *E*. *coli* DH5α cells and purified using glutathione-Sepharose beads (GE Healthcare) in STET buffer (10 mM Tris-HCl pH 8.0, 100 mM NaCl, 1 mM EDTA, 5% Triton X-100, 2 mM DTT, 1 mM PMSF, 1 mM benzamidine, 2 mg ml^-1^ leupeptin, 2 mg ml^-1^ pepstatin). Hog1 (1 μg) was pre-activated with 0.5 μg of Pbs2^EE^ in the presence of kinase buffer (50 mM Tris-HCl pH 7.5, 10 mM MgCl_2_, 2 mM DTT) and 50 μM of ATP. After 20–30 minutes incubation at 30°C, eluted wild-type Spt4 or mutant Spt4^T42AS43A^ were added to the pre-activated mixture together with [γ-^32^P]ATP (0.1 mCi/ml) and incubated for 30 minutes at 30°C. The reaction was terminated by adding 10 μl of 5X Laemli buffer and boiling the samples for 5 minutes. Labeled proteins were resolved by SDS-PAGE and detected by autoradiography. Total protein level was detected by staining with Coomassie Brilliant Blue.

### *In vivo* phosphorylation assay

TAP-tagged Spt4 and Spt4^T42AS43A^ cells were grown to mid-log phase on YPD and were then subjected to a brief osmostress (0.4 M NaCl for 5 minutes) or were left untreated. For each condition, 100 ml aliquots were harvested by centrifugation and pellets were resuspended in 300 μl of buffer A (50 mM Tris-HCl pH 8.0, 15 mM EDTA, 15 mM EGTA, 0.1% Triton X-100 ± 150 mM NaCl) supplemented with protease and phosphatase inhibitors (1 mM PMSF, 2 μg ml^-1^ leupeptin, 2 μg ml^-1^ pepstatin, 1 mM benzamidine, 2 mM DTT, 10 mM sodium fluoride, 25 mM β-glycerophosphate, 1 mM sodium orthovanadate, 1 mM sodium pyrophosphate). Chilled glass beads were added, and cells were disrupted with a Fast-prep glass bead beater (Millipore). Protein extracts (10 mg) were incubated with rabbit IgG agarose (Sigma) beads for 2–3 hours at 4°C. Beads were washed 10 times with supplemented buffer A + NaCl, and once with 50 mM Tris-HCl pH 6.8, boiled in SDS-loading buffer for 5 minutes and analyzed by 11% SDS-PAGE. Proteins were then transferred to PVDF membranes and phosphorylation was detected by immunoblotting with an anti-phospho Ser/Thr antibody (BD Transduction Laboratories).

### Northern blot analysis

Yeast strains were grown to mid-log phase in rich medium, immediately subjected or not to osmostress (0.4 M NaCl), and samples were taken at the indicated times. Galactose induction was performed by growing cells on YP 2% raffinose up to mid-log phase and shifting them to 2% galactose for the indicated times. For the expression analysis of genes under the control of the LexA promoter as well as for the GLAM assays, mid-log phase cells were grown in minimal medium for plasmid maintenance. Total RNA and Northern hybridization analyses were carried out according to standard procedures and expression of specific genes was detected by hybridization of the Northern blot membranes with labeled PCR fragments of *CTT1* (1.7 kbp), *STL1* (0.88 kbp), *ALD3* (0.6 kbp), *GRE2* (0.9 kbp), *ENO1* (1.3 kbp), and *GAL1* (0.88 kbp). Signals were quantified using a Typhoon 8600 phosphorimager (Molecular Dynamics) and autoradiographs were obtained on Carestream Kodak Biomax XAR film (Sigma). In the case of the GLAM assay, the phosphorimaging analyses were performed in a STORM-840 imaging system (GE Healthcare) and quantified using GelQuant.NET software provided by biochemlabsolutions.com.

### RNA sequencing and analysis

Yeast strains were cultured to mid-log phase in rich medium and then subjected or not to osmostress (0.4 M NaCl) for 15 minutes. Three biological replicates for each condition were performed. Total RNA was extracted by using the standard hot phenol and glass-beads protocol. Libraries were prepared using the TruSeq stranded mRNA sample preparation kit v2 (Illumina) according to the manufacturer’s protocol, and starting with 1 μg of total RNA for poly(A)-mRNA selection. Libraries were first analyzed on an Agilent Bioanalyzer using a DNA 1000 chip to estimate the quantity and check the size distribution. Libraries were then quantified by qPCR using the KAPA library quantification kit (KapaBiosystems) prior to sequencing with 50 bp single-end reads on Illumina's HiSeq 2500 with v4 sequencing chemistry. All RNA-seq experiments were run in triplicates. Quality control of raw sequencing reads was performed by FASTQC; all samples passed the imposed quality restrictions and were used for further analysis. RNA-seq reads were mapped to the sacCer3 reference genome (obtained via UCSC goldenPath) using TopHat2 v2.1.0 [48] with default parameters. Quantification was performed by featureCounts v1.5.1 [49] using the Ensembl R64-1-1 annotation and only considering uniquely mapping reads. Subsequent analyses were conducted using the statistical programming language R, and the DESeq2 package (v.1.14.1; [50]) was used for library size normalization (RLE) and differential expression testing. Osmo-responsive genes were defined as genes that significantly exceeded an absolute log2-fold change (log2FC) of 1 after multiple testing correction (FDR < 0.05). In contrast, osmo-unchanged genes were defined as genes whose absolute log2FC was not significantly larger than 0.5 (FDR < 0.05). Results were visualized using the ggplot2 R-package (ggplot2.org). Data have been deposited in the Gene Expression Omnibus (GEO) database (GSE98352).

### Chromatin immunoprecipitation (ChIP) assays

ChIP was performed essentially as previously described [16]. Yeast cultures were grown in rich YPD medium to mid-log phase before exposing them to osmostress (0.4 M NaCl) and samples were taken at the times specified. Anti-Rpb1 (8WG16, Covance) or anti-phospho RNA Polymerase II (S2) (Bethyl Laboratories; Cat. Number A300-654) antibodies were used for RNA Pol II ChIPs. Anti-Rpb3 (ab81859, Abcam) monoclonal antibody was used for GLAM ChIPs. Anti-Myc (9E10 hybridoma) was used for Spt5-Myc binding. Rabbit IgGs were used for TAP-tagged versions of wild-type Spt4 or mutated Spt4^T42AS43A^ binding. Oligonucleotides to amplify regions of *CTT1* (-432/-302 for the promoter; +737/+836 for the ORF), *STL1* (-682/-583 for the promoter; +1477/+1575 for the ORF) and *TEL1* (a telomeric region on the right arm of chromosome VI that was used as an internal normalizing control sequence for each DNA analyzed; TEL RTa: ACCACTCAAAGAGAAATTTACTGGAAGA and TEL RTb: CTCGTTAGGATC ACGTTCGAATC) were used for real-time PCR analysis with Power SYBR Green PCR Master Mix (Applied Biosystems), employing an Applied Biosystems ViiA7 detector. Locations are indicated by the distance respect to the correspondent ATG initiation codon. In GLAM ChIPs the primers used correspond to 0, +900, +1900, +3000, +4000 and +4700 after the ATG initiation codon of the *STL1-lacZ* transcription unit.

### Co-precipitation assays

To study Spt4 and Spt5 interaction, cells expressing a 9myc-tagged version of Spt5 and TAP-tagged versions of either wild-type Spt4 (YSC30) or mutated Spt4^T42AS43A^ (YSC31), as well as cells with only Spt5-9myc (YSC29) as a negative control for TAP pull down, were grown to mid-log phase on YPD, subjected or not to osmostress (0.4 M NaCl for 10 minutes) and harvested at 4°C. Protein extracts and pull down of TAP-tagged proteins (5 mg) were performed as for the *in vivo* phosphorylation assays. Proteins were then transferred to PVDF membranes and Spt5-9myc was detected by immunoblotting with an anti-myc monoclonal antibody (9E10 hybridoma). For analysis of Spt4 and RNA Pol II interaction, we used cells expressing the TAP-tagged versions of wild-type Spt4 (YAS3) or mutated Spt4^T42AS43A^ (YAS13), as well as BY4741, which was used as the negative control for TAP pull down. Osmostress and TAP purification was performed as indicated above and RNA Pol II was detected using an anti-Rpb1 (8WG16, Covance) monoclonal antibody.

### Growth phenotype analysis

Yeast cultures were grown to mid-log phase in YPD and then diluted to OD_660_ = 0.05 in YPD, 1.4 M KCl or 1.4 M NaCl. The analyses were performed in quadruplicate. Cell growth was monitored (30°C, constant agitation, 1.5-hour time points) at OD_660_ for 21 hours. Growth curves were analyzed using a Synergy H1 Hybrid Reader (BioTek).

### Spot test

Yeast cultures were grown to mid-log phase in YPD, then diluted to OD_600_ = 0.05 and cell suspensions were diluted using 10-fold serial dilutions in 96-well microtiter plates (200 μl/well). The cells were spotted onto YPD and SC plates containing 6-azauracil and growth was monitored and photographed at appropriate time points.

### Sequence alignments

Spt4 protein sequence alignments were made with Geneious software version 7.1.9 (http://www.geneious.com, [51]).

### Statistical analysis

Data are reported as mean ± SD. Statistical significance was assessed using a Student’s t test for equality of means, two-tailed and equal variance assumed.

## Supporting information

S1 Fig List of the 40 TAP-tagged proteins involved in transcriptional elongation that were subjected to an *in vitro* phosphorylation assay with active Hog1. (PDF)

S2 Fig **(A)** Comparison of the osmo-responsive gene expression between the untagged and TAP-tagged Spt4 strains, in the absence of osmostress (0) and after treatment with 0.4 M NaCl for 5, 10 and 20 min. Northern blotting was performed with radiolabeled probes specific for *CTT1*, *STL1* and *ENO1* (as a loading control). **(B)** Quantification of relative gene expression levels of *CTT1* and *STL1* from (A) is shown. Results are expressed as the percentage of the ratio with *ENO1* expression, normalized to the maximum level of expression in the untagged wild type which is shown as 100%. Data of three biological replicates were used and means ± SD were calculated. t-test analysis was calculated. ns means p≥0.1. **(C)** Spt4-TAP, Spt4^T42AS43A^-TAP and Spt5-Myc protein expression was analyzed by Western blotting from TCA extracts of Spt4 wild type and Spt4^T42AS43A^ mutant strains. Quantification using ImageJ software of relative protein expression levels of Spt4-TAP and Spt5-Myc is shown.
(PDF)

S3 Fig Volcano plot showing significantly altered gene expression in wild type (Spt4 WT) strain upon stress (0.4 M NaCl, 15 min). Dashed vertical lines indicate a 2-fold change in expression (up-/down-regulation), whereas the dashed horizontal line indicates an adjusted p-value of 0.05. n indicated the number of genes significantly altered in osmostress compared to non-stress conditions.
(PDF)

S4 Fig Significance (Student’s t test) of the association of RNA Pol II at *STL1-lacZ* monitored by ChIP using the monoclonal anti-Rpb3 antibody (ab81859; Abcam) in wild type (wt; blue), Spt4^T42AS43A^ (red) and *spt4Δ* (green) cells upon osmostress (7.5 minutes after 0.4 M NaCl addition). Asterisks located immediately above the red and green bars indicate the difference significance of the means of the respective mutant in that amplicon *versus* the wild type strains, which is represented in the center for easier comparison. Significant differences between neighboring amplicons for each strain are indicated in upper brackets. *, p<0.05; **, p<0.01.
(PDF)
