# Supplementary material for: Regulation of transcription elongation in response to osmostress
Source: PLoS Genet. 2017 Nov 20;13(11):e1007090. doi: 10.1371/journal.pgen.1007090 (PMC5720810; doi:10.1371/journal.pgen.1007090)
Supplement: S1 Fig — (PDF) [file pgen.1007090.s001.pdf]

Figure S1

YBR279W	PAF1
YCR093W	CDC39
YDR138W	HPR1
YER169W	RPH1
YGL043W	DST1
YGL207W	SPT16
YGL244W	RTF1
YGL246C	RAI1
YGR005C	TFG2
YGR063C	SPT4
YGR116W	SPT6
YGR134W	CAF130
YGR186W	TFG1
YGR252W	GCN5
YHR167W	THP2
YIL038C	NOT3
YJL006C	CTK2
YJL115W	ASF1
YJL179W	PFD1
YKL005C	BYE1

YKL139W	CTK1
YKL160W	ELF1
YKR048C	NAP1
YLR200W	YKE2
YLR418C	CDC73
YML010W	SPT5
YML062C	MFT1
YML069W	POB3
YML094W	GIM5
YML112W	CTK3
YMR039C	SUB1
YNL068C	FKH2
YNL139C	THO2
YNL153C	GIM3
YNL288W	CAF40
YOL145C	CTR9
YOR048C	RAT1
YOR123C	LEO1
YPR072W	NOT5
YPR133C	SPN1
