# Supplementary figures and images for: Regulation of transcription elongation in response to osmostress
Source: PLoS Genet. 2017 Nov 20;13(11):e1007090. doi: 10.1371/journal.pgen.1007090 (PMC5720810; doi:10.1371/journal.pgen.1007090)

Figure S2

A

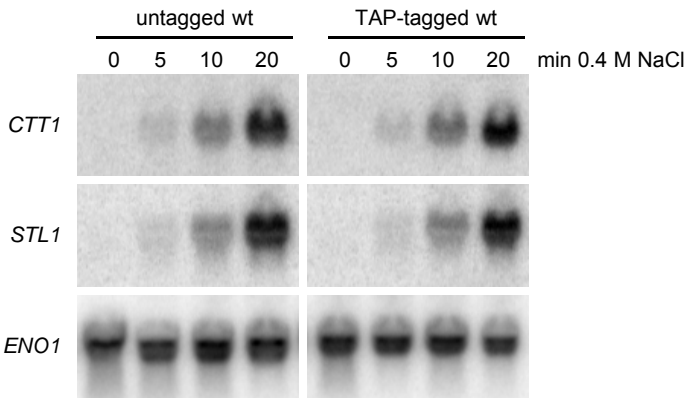

B

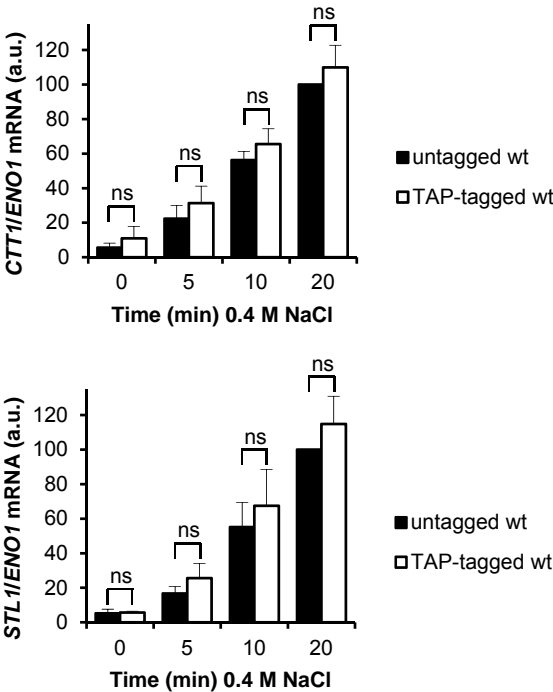

C

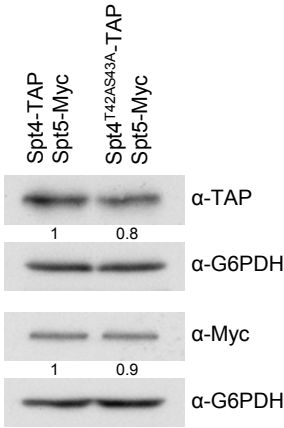

Supplement: S2 Fig — (A) Comparison of the osmo-responsive gene expression between the untagged and TAP-tagged Spt4 strains, in the absence of osmostress (0) and after treatment with 0.4 M NaCl for 5, 10 and 20 min. Northern blotting was performed with radiolabeled probes specific for CTT1, STL1 and ENO1 (as a loading control). (B) Quantification of relative gene expression levels of CTT1 and STL1 from (A) is shown. Results are expressed as the percentage of the ratio with ENO1 expression, normalized to the maximum level of expression in the untagged wild type which is shown as 100%. Data of three biological replicates were used and means ± SD were calculated. t-test analysis was calculated. ns means p≥0.1. (C) Spt4-TAP, Spt4T42AS43A-TAP and Spt5-Myc protein expression was analyzed by Western blotting from TCA extracts of Spt4 wild type and Spt4T42AS43A mutant strains. Quantification using ImageJ software of relative protein expression levels of Spt4-TAP and Spt5-Myc is shown. (PDF) [file pgen.1007090.s002.pdf]

Figure S3

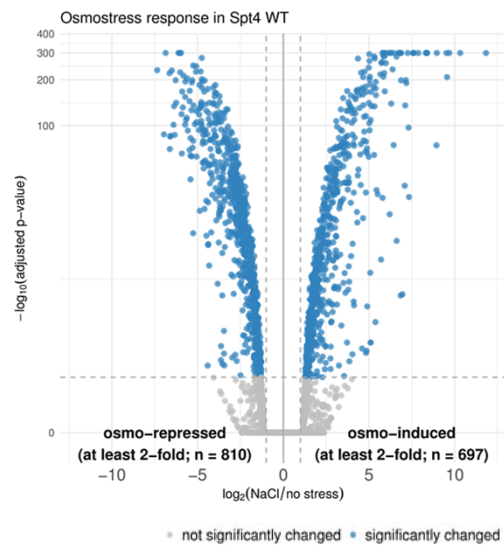

Supplement: S3 Fig — Dashed vertical lines indicate a 2-fold change in expression (up-/down-regulation), whereas the dashed horizontal line indicates an adjusted p-value of 0.05. n indicated the number of genes significantly altered in osmostress compared to non-stress conditions. (PDF) [file pgen.1007090.s003.pdf]

Figure S4

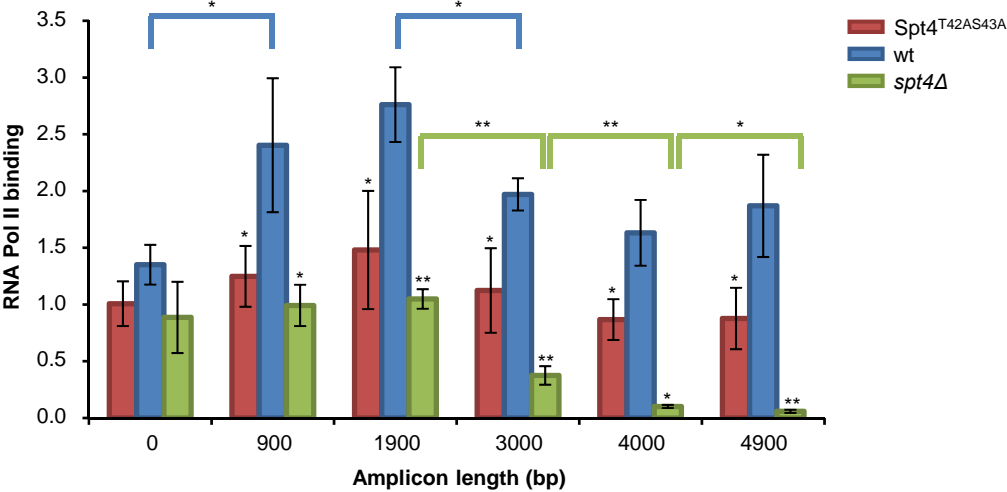

Supplement: S4 Fig — Asterisks located immediately above the red and green bars indicate the difference significance of the means of the respective mutant in that amplicon versus the wild type strains, which is represented in the center for easier comparison. Significant differences between neighboring amplicons for each strain are indicated in upper brackets. *, p<0.05; **, p<0.01. (PDF) [file pgen.1007090.s004.pdf]
